# Serum exosomal long noncoding RNA CRNDE as a prognostic biomarker for hepatocellular carcinoma

**DOI:** 10.1002/jcla.23959

**Published:** 2021-10-06

**Authors:** Ting Wang, Hengkai Zhu, Min Xiao, Shao Zhou

**Affiliations:** ^1^ Department of Hepatobiliary and Pancreatic Surgery Shulan (Hangzhou) Hospital Affiliated to Zhejiang Shuren University Shulan International Medical College Hangzhou China; ^2^ Department of Hepatobiliary and Pancreatic Surgery The First Affiliated Hospital of Zhejiang University School of Medicine Hangzhou China

**Keywords:** biomarker, CRNDE, exosome, hepatocellular carcinoma, prognosis

## Abstract

**Background:**

Accumulating evidence has shown that long noncoding RNA (lncRNA) CRNDE functions as an oncogene in many cancer types. However, its clinical value has not yet been explored in hepatocellular carcinoma (HCC).

**Methods:**

A total of 166 patients with HCC and 100 healthy volunteers were enrolled in this study. The expression levels of serum exosomal lncRNA CRNDE were detected in patients with HCC and controls by quantitative real‐time PCR (qRT‐PCR).

**Results:**

The serum exosomal lncRNA CRNDE expression levels were significantly increased in patients with HCC compared with normal controls. High serum exosomal lncRNA CRNDE expression was significantly associated with tumor size, tumor differentiation, and TNM stage. Receiver operating characteristic (ROC) analysis revealed that an area under the ROC curve (AUC) of 0.839, with a sensitivity and specificity of 69.3% and 85.0%. In addition, the overall survival (OS) and disease‐free survival (DFS) were significantly longer in patients with lower serum exosomal lncRNA CRNDE expression compared to those with higher CRNDE expression. Moreover, HCC patients with cirrhosis had worse OS and DFS than those without cirrhosis. Univariate and multivariate analyses indicated that high serum exosomal lncRNA CRNDE expression was an independent indicator of poor prognosis.

**Conclusion:**

Taken together, serum exosomal lncRNA CRNDE might serve as a potential biomarker for HCC diagnosis and prognosis.

## Graphical abstract

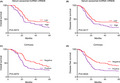

## 1 INTRODUCTION

In 2018, liver cancer is one of the most common cancers in China with about 3,68,960 cancer‐related deaths and has become a major public health problem in the country.^1^ The majority of liver cancer (75%–80%) is hepatocellular carcinoma (HCC).^2^ Though great improvements in treatment strategies have been made in the past decades, the long‐term survival rate of patients with HCC remains very unfavorable mainly because of late diagnosis, cancer recurrence, and tumor metastasis.^3^, ^4^ Treatment of surgical resection is usually used for early‐stage HCC. However, most patients are diagnosed at advanced stages due to the lack of reliable markers for early detection.^5^ Therefore, to improve the outcome of patients with HCC, the identification of novel biomarkers for predicting the recurrence and prognosis of this disease is urgently required.

Long noncoding RNAs (lncRNAs) are a class of transcripts longer than 200 nucleotides and are involved in a variety of cellular processes including cell proliferation, apoptosis, and differentiation.^6^, ^7^, ^8^ LncRNAs actively participate in the initiation and progression of different cancer types including HCC. For instance, TGLC15 was significantly upregulated in HCC, and TGLC15 overexpression predicted unfavorable clinical outcome. Mechanistically, increased TGLC15 promoted the oncogenic activities of HCC cells in vitro and in vivo through interacting with SOX4.^9^ Similarly, a novel lncRNA termed HLNC1 was demonstrated to promote tumorigenesis of HCC by interacting with USP49.^10^ Exosomes are membrane vesicles with a size range of 30–150 nm and can be found in serum, plasma, urine, saliva, and breast milk. Exosomes contain various types of nucleic acids, such as miRNAs, proteins, and lncRNAs.^11^, ^12^ lncRNAs in serum exosomes can be stably detected and emerged as candidate biomarkers for the detection of HCC.

The colorectal neoplasia differentially expressed (CRNDE) gene locates on chromosome 16, and has been reported as an oncogene in several malignancies, such as colorectal cancer,^13^ non‐small cell lung cancer,^14^ cervical cancer,^15^ pancreatic cancer,^16^ and papillary thyroid cancer.^17^ However, to the best of our knowledge, the clinical significance of lncRNA CRNDE in serum exosomes of patients with HCC has not been analyzed. In this study, the lncRNA CRNDE expression levels in exosomes isolated from the serum samples of patients with HCC and healthy volunteers were detected. The application significance of serum exosomal lncRNA CRNDE as a biomarker for the detection and prognosis of HCC was assessed.

## 2 MATERIALS AND METHODS

### 2.1 Ethical approval

Signed informed consents were collected from all participants prior to the recruitment. The study protocol was approved by the Ethics Committee of Shulan International Medical College.

### 2.2 Sample collection

The current study enrolled 166 patients diagnosed with HCC and 100 healthy volunteers as controls. The diagnosis of HCC was based on World Health Organization (WHO) criteria. None of patients received any radiotherapy or chemotherapy before blood samples collection. Patients with HCC were staged by the TNM classification of the International Union against Cancer (Sixth Edition). The clinical data included age, sex, AFP, cirrhosis, hepatitis B, tumor number, tumor size, tumor differentiation, and tumor stage were presented in Table 1. Overall survival (OS) was calculated as the time from diagnosis to death or the last observation point. Disease‐free survival (DFS) was calculated as the time from diagnosis to relapse or the last observation point.

**TABLE 1:** Association between CRNDE expression and clinical parameters in HCC patients

Clinical characteristics	Number	Serum exosomal lncRNA CRNDE		*p*‐value
		High	Low	
Age, years				
<50	48	22	26	0.4935
≥50	118	61	57	
Sex				
Male	143	73	70	0.5003
Female	23	10	13	
AFP, ng/μl				
<20	71	31	40	0.1580
≥20	95	52	43	
Cirrhosis				
Negative	64	28	36	0.2021
Positive	102	55	47	
Hepatitis B				
Negative	51	22	29	0.2389
Positive	115	61	54	
Tumor number				
Single	108	51	57	0.3287
Multiple	58	32	26	
Tumor size, cm				
<5	94	35	59	0.0002
≥5	72	48	24	
Tumor differentiation				
Well	104	43	61	0.0039
Moderate/poor	62	40	22	
TNM stage				
I/II	64	21	43	0.0005
III/IV	102	62	40	

### 2.3 Exosomal isolation

After blood was drawn from patients with HCC and healthy volunteers, serum was immediately isolated by centrifugation at 2000 g for 10 min at room temperature and then stored at −80°C until further use. Exosomes were isolated from serum using ExoQuick Exosome Precipitation Solution (System Biosciences). Briefly, serum was centrifuged at 3000 × g for 15 min and filtrated with a 0.22 μm syringe filter (EMD Millipore) to remove cell debris. Then, serum supernatant was mixed with one‐fourth volume of ExoQuick solution. The mixture was centrifuged at 1500 g for 30 min after incubation at 4°C overnight. The final pellets containing exosome fractions weres resuspended in PBS.

### 2.4 Quantitative real‐time PCR

The total RNA was extracted from the serum using an miRNeasy Serum/Plasma kit (Qiagen). In the RNA isolation step, 2 μl synthetic Caenorhabditis elegans cel‐miR‐39 (RiboBio) was added as a spike‐in control. For cDNA synthesis, TaqMan MicroRNA Reverse Transcription Kit (Applied Biosystems, Thermo Fisher Scientific) was performed. qRT‐PCR was carried out using SYBR Premix DimerEraser kit (TaKaRa) on an ABI PRISM 7900 Sequence Detection System (Applied Biosystems). Each experiment was repeated in triplicate. The relative expression levels of serum exosomal CRNDE were normalized against cel‐miR‐39 using the 2^–ΔΔCt^ method.

### 2.5 Statistical analysis

All statistical calculations were performed using GraphPad Prism 9.0 (GraphPad Software) and MedCalc 16.4.3 (MedCalc). The significance of serum exosomal lncRNA CRNDE between the two groups was evaluated by Mann‐Whitney *U* test. Chi‐square test was used to analyze the categorical data. Diagnostic power of serum exosomal lncRNA CRNDE was assessed by receiver operating characteristic (ROC) curves, and the area under the curve (AUC) was calculated. Cox proportional‐hazards regression analysis was performed to analyze univariate and multivariate risk ratios for OS. OS and DFS were drawn and compared using the Kaplan‐Meier method plus the log‐rank test. *p* values less than 0.05 were considered statistically significant.

## 3 RESULTS

### 3.1 Serum exosomal lncRNA CRNDE was highly expressed in HCC

**FIGURE 1:**
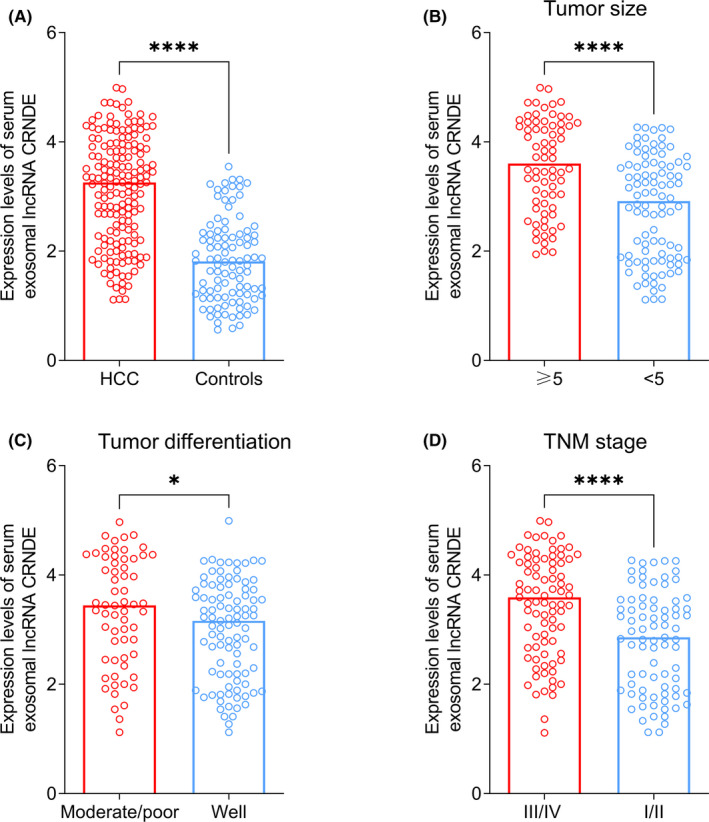
(A) Comparison of serum exosomal lncRNA CRNDE levels between HCC patients and normal controls. (B) Serum exosomal lncRNA CRNDE levels in HCC patients with different tumor size. (C) Serum exosomal lncRNA CRNDE levels in HCC patients with different tumor differentiation status. (D) Serum exosomal lncRNA CRNDE levels in HCC patients at different tumor stage

The levels of serum exosomal lncRNA CRNDE were markedly higher in patients with HCC compared with normal controls (****p < 0.0001, Figure 1A). In addition, increased serum exosomal lncRNA CRNDE levels were highly associated with HCC patients with ≥5 tumor size (****p < 0.0001, Figure 1B). Moreover, HCC patients with moderate/poor differentiation had higher serum exosomal lncRNA CRNDE expression than those with well differentiation (*p = 0.0219, Figure 1C). Furthermore, a significant increase in serum exosomal lncRNA CRNDE expression was observed in stage III/IV patients in comparison with stage I/II patients (****p < 0.0001, Figure 1D).

### 3.2 Relationship between serum exosomal lncRNA CRNDE expression and clinical parameters

According to the median serum exosomal lncRNA CRNDE expression, 58 cases were categorized into the high serum exosomal lncRNA CRNDE group, while the remaining 58 patients were in the low serum exosomal lncRNA CRNDE group. As shown in Table 1, high serum exosomal lncRNA CRNDE expression was strongly associated with tumor size (*p* = 0.0002), tumor differentiation (*p* = 0.0039), and TNM stage (*p* = 0.0005). However, no significant correlation existed between serum exosomal lncRNA CRNDE expression and other clinical characteristics such as age, sex, AFP, cirrhosis, hepatitis B, and tumor number (all *p* > 0.05).

### 3.3 The diagnostic efficiency of serum exosomal lncRNA CRNDE for HCC

**FIGURE 2:**
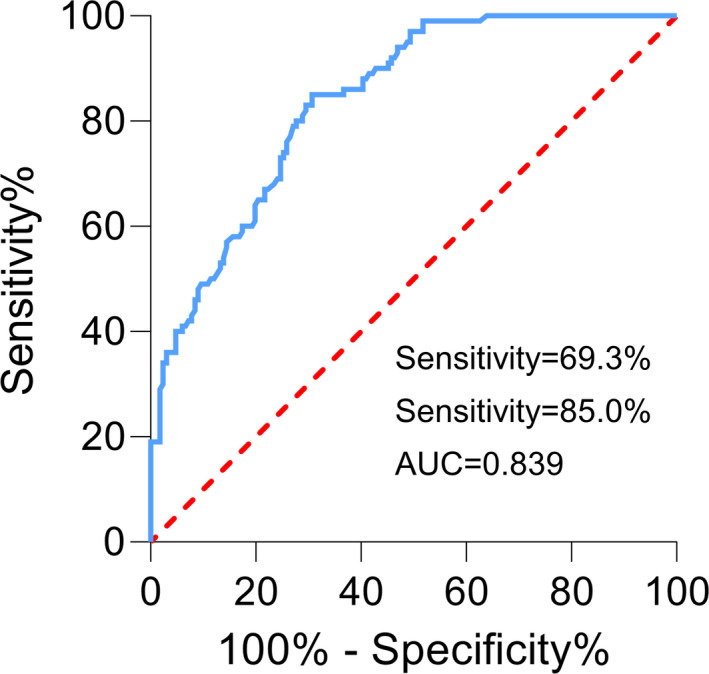
Serum exosomal lncRNA CRNDE could well identify HCC patients from controls

Receiver operating characteristic curve analysis was performed to analyze the efficiency of serum exosomal lncRNA CRNDE as a diagnostic indicator for HCC detection. Figure 2 demonstrated that the sensitivity was 69.3% and the specificity was 85.0% with an AUC of 0.839 (95% CI = 0.794–0.885), indicating that serum exosomal lncRNA CRNDE could well identify HCC cases from normal controls.

### 3.4 Relationship between serum exosomal lncRNA CRNDE expression and HCC prognosis

**FIGURE 3:**
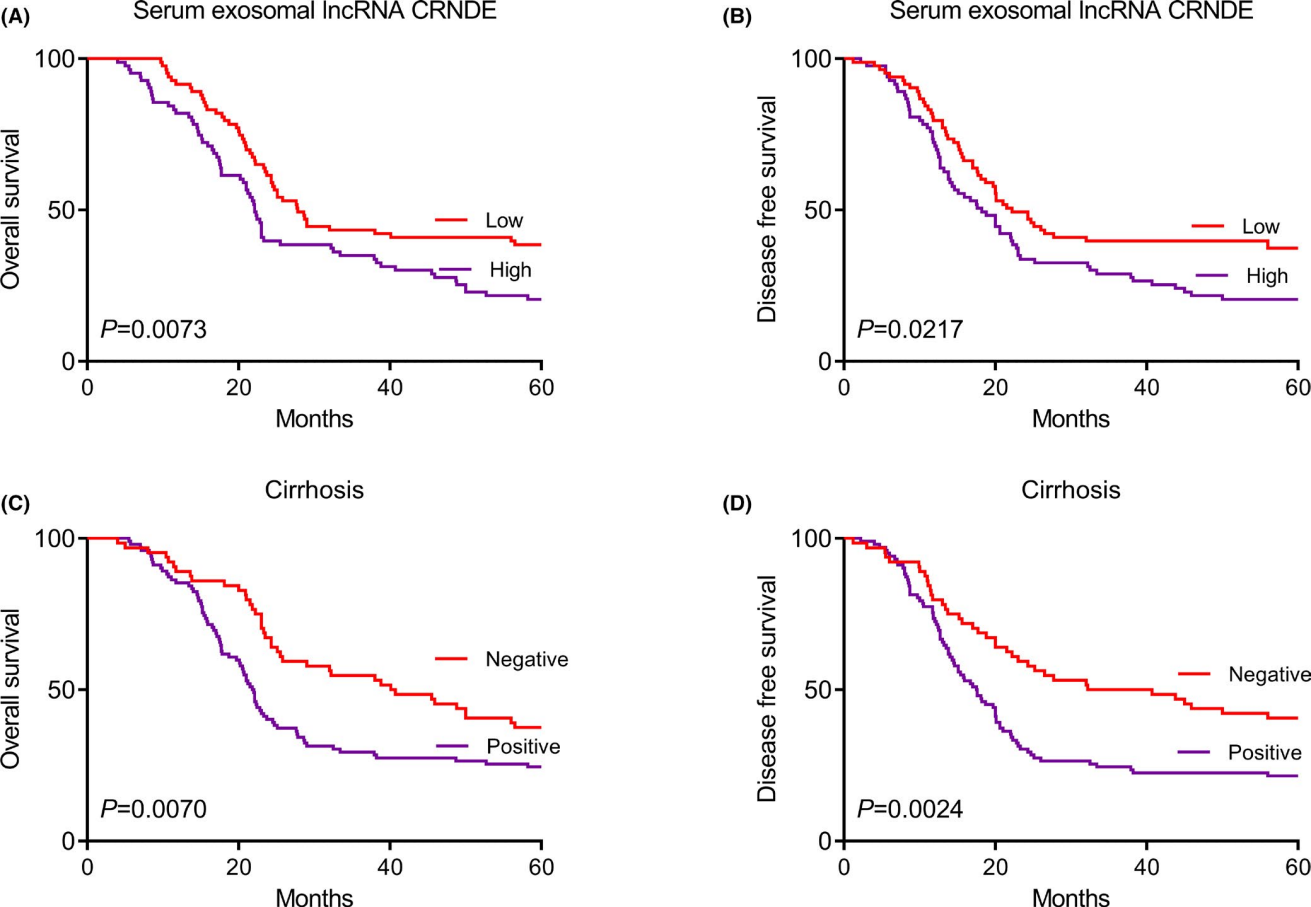
(A) OS curve of HCC patients stratified by serum exosomal lncRNA CRNDE expression. (B) DFS curve of HCC patients stratified by serum exosomal lncRNA CRNDE expression. (C) OS curve of HCC patients stratified by cirrhosis. (D) DFS curve of HCC patients stratified by cirrhosis

The Kaplan‐Meier method was used to plot OS and DFS according to the serum exosomal lncRNA CRNDE levels. Patients with HCC in high serum exosomal lncRNA CRNDE expression group had significantly shorter OS (*p* = 0.0073, Figure 3A) and DFS (*p* = 0.0217, Figure 3B). Then, OS and DFS curves of patients with HCC stratified by cirrhosis were also plotted. The subgroup of patients with cirrhosis had significant shorter OS (*p* = 0.0070, Figure 3C) and DFS (*p* = 0.0024, Figure 3D) compared to those without cirrhosis.

To assess whether serum exosomal lncRNA CRNDE might serve as an independent prognostic marker for OS and DFS in HCC, the effects of serum exosomal lncRNA CRNDE expression levels and prognosis of patients was evaluated. As shown in Table 2, the results revealed that serum exosomal lncRNA CRNDE (RR = 3.81, 95% CI = 1.81–6.74, *p* = 0.005), tumor size (RR = 2.91, 95% CI = 1.12–4.73, *p* = 0.032), tumor differentiation (RR = 3.28, 95% CI = 1.96–6.27, *p* = 0.024), and TNM stage (RR = 4.60, 95% CI = 2.30–8.26, *p* < 0.001) were independent prognostic markers for OS. Moreover, the multivariate regression analysis indicated that serum exosomal lncRNA CRNDE (RR = 3.25, 95% CI = 1.65–6.06, *p* = 0.012), tumor differentiation (RR = 3.01, 95% CI = 1.58–5.41, *p* = 0.026), and TNM stage (RR = 4.20, 95% CI = 2.11–7.08, *p* = 0.006) were independent prognostic factors affecting DFS of patients with HCC.

**TABLE 2:** Univariate and multivariate analyses for OS by Cox regression model

Factors	Univariate analysis			Multivariate analysis		
	RR	95% CI	*p*‐value	RR	95% CI	*p*‐value
Tumor size	2.91	1.12–4.73	0.032	1.71	0.91–3.14	0.091
Tumor differentiation	3.28	1.96–6.27	0.024	3.01	1.58–5.41	0.026
TNM stage	4.60	2.30–8.26	<0.001	4.20	2.11–7.08	0.006
Serum exosomal lncRNA CRNDE	3.81	1.81–6.74	0.005	3.25	1.65–6.06	0.012

Abbreviations: CI, confidence interval; RR, risk ratio.

## 4 DISCUSSION

Previous studies have reported that lncRNA CRNDE enhances the migration, viability, and invasive ability of HCC cells. For instance, CRNDE expression was significantly elevated in the cancerous tissues and cell lines of HCC, and inhibition of CRNDE dramatically suppressed HCC pathogenesis and promoted HCC cell chemosensitivity.^18^ CRNDE overexpression significantly enhanced HCC cell proliferation, migration, and invasion capacities by inversely regulating miR‐384 or miR‐217 expression, while downregulation of CRNDE exhibited the opposite effects.^19^, ^20^ CRNDE silencing greatly suppressed HCC cell migration, invasive capacity, cell epithelial‐mesenchymal transition (EMT) process in vitro, and tumor growth in vivo.^21^ The data indicated than CRNDE might function as an oncogene in this malignancy.

In this study, the results showed that serum exosomal lncRNA CRNDE expression was significantly upregulated compared with that of healthy volunteers. Serum exosomal lncRNA CRNDE overexpression occurred more frequently in HCC patients with larger tumor size, poorer differentiation, and advanced TNM stage. In addition, serum exosomal lncRNA CRNDE expression showed good performance to differentiate patients with HCC from normal controls. High serum exosomal lncRNA CRNDE expression was closely associated with aggressive clinical parameters. The OS and DFS rate of HCC patients with high serum exosomal lncRNA CRNDE expression was markedly worse than those with lower CRNDE expression. Similarly, HCC patients with cirrhosis had significant shorter OS and DFS than those without cirrhosis. Furthermore, univariate and multivariate analyses revealed that serum exosomal lncRNA CRNDE was an independent marker for OS. Thus, serum exosomal lncRNA CRNDE might be a valuable biomarker to predict the prognosis in patients with HCC.

LncRNA CRNDE upregulation was also found in colorectal cancer (CRC) tissues and associated with poor clinical variables. Downregulation of lncRNA CRNDE markedly inhibited cell proliferation, induced cell apoptosis, and reduced chemoresistance by targeting miR‐181a‐5p.^13^, ^22^ CRNDE expression was significantly elevated both in tissues and cell lines of non‐small cell lung cancer (NSCLC). NSCLC patients with increased CRNDE expression predicted a worse prognosis. CRNDE inhibition remarkably attenuated NSCLC cell proliferation, colony formation, migration, stimulated cell apoptosis in vitro and suppressed the tumorigenesis in vivo through regulating miR‐338‐3p and miR‐641.^14^, ^23^ In addition, CRNDE expression was highly expressed in cervical cancer (CC) tissues and cells. Enforced CRNDE expression significantly enhanced CC cell proliferation, migration, and invasion, while CRNDE inhibition markedly suppressed the cancer progression by upregulating miR‐4262 or PUMA expression.^15^, ^24^ Moreover, CRNDE upregulation was not only observed both in cancerous tissues and cell lines of pancreatic cancer (PC), but also strongly associated with worse clinical variables, including differentiation, TNM stage, and lymph node metastasis. CRNDE silencing greatly decreased PC cell proliferation, migration, and invasion in vitro and in vivo.^16^ Furthermore, CRNDE expression was significantly upregulated in papillary thyroid cancer (PTC) tissues and cell lines. Ectopic CRNDE expression significantly stimulated PTC cell proliferation and invasion trough silencing miR‐384 expression, while CRNDE downregulation exerted tumor‐suppressive functions.^17^Although serum exosomal lncRNA CRNDE is promising for predicting the prognosis of HNSCC, it should be noted that HCC is a very complicated disease. Therefore, combining serum exosomal lncRNA CRNDE with traditional molecular biomarkers or other newly discovered signatures is of great significance for improving the clinical outcome of HCC. For instance, Jiang et al^25^ recently developed a robust four‐lncRNA signature for evaluating the prognostic risk for HCC.

## 5 CONCLUSIONS

In conclusion, the study highlighted the potential role of serum exosomal lncRNA CRNDE for the diagnosis and prognosis of HCC. The results demonstrated that serum exosomal lncRNA CRNDE could serve as a novel predictive biomarker for the prognosis of HCC. The limitation of the study is the relatively small sample size, and future studies with a larger sample size will be required.

## CONFLICT OF INTEREST

No competing interests.

## INFORMED CONSENT

Signed informed consent was collected from all participants prior to the recruitment.

## Data Availability

The datasets used and/or analyzed during the current study are available from the corresponding author on reasonable request.
